# Loading monocytes with magnetic nanoparticles enables their magnetic control without toxicity

**DOI:** 10.3389/fbioe.2024.1498120

**Published:** 2025-01-08

**Authors:** Laura Mödl, Lucas R. Carnell, Rene Stein, Andrea Kerpes, Felix Pfister, Barbara Wirthl, Wolfgang A. Wall, Christoph Alexiou, Christina Janko

**Affiliations:** ^1^ Department of Otorhinolaryngology, Head and Neck Surgery, Section of Experimental Oncology and Nanomedicine (SEON), Else Kröner-Fresenius-Stiftung Professorship, Universitätsklinikum Erlangen, Erlangen, Germany; ^2^ Friedrich-Alexander-Universität Erlangen-Nürnberg (FAU), Erlangen, Germany; ^3^ Department of Engineering Physics and Computation, TUM School of Engineering and Design, Institute for Computational Mechanics, Technical University of Munich, Garching bei München, Germany

**Keywords:** iron oxide nanoparticles, computer simulation, magnetic cell targeting, nanomedicine, solid cancer

## Abstract

**Background:**

With the help of superparamagnetic iron oxide nanoparticles (SPIONs), cells can be magnetically directed so that they can be accumulated at target sites. This principle can be used to make monocytes magnetically steerable in order to improve tumor accumulation, e.g., for immunotherapy with chimeric antigen receptor (CAR) monocytes. Here, we investigated the loading of monocytic THP-1 cells with SPIONs, analyzed their impact on the viability and cellular reactive oxygen species (ROS) generation and their magnetic enrichment. Finally, we compared and confirmed the experimentally generated magnetic enrichment data with computational simulations.

**Methods and results:**

When THP-1 cells were incubated with citrate-coated SPIONs (SPION^Citrate^) or citrate-stabilized gold-coated SPIONs (SPION^Gold^), cells ingested the particles, as determined via transmission electron microscopy and atomic emission spectroscopy. Flow cytometry showed that the particles were biocompatible and produced hardly any ROS. With SPION-loading, cells accumulated in Ibidi flow slides at the edge of a Neodym magnet, where the magnetic field and force were maximal, as calculated by our computational model.

**Conclusion:**

THP-1 cells were successfully loaded with SPIONs, which exhibited excellent biocompatibility and provided the cells with magnetic steerability. The computational model predicted the actual magnetic accumulation of the SPION-loaded cells, enabling a more systematic and faster exploration of the design space in the future.

## 1 Introduction

Superparamagnetic iron oxide nanoparticles (SPIONs) have been exploited in medicine for several purposes such as contrast agents in magnetic resonance imaging (MRI), for magnetic hyperthermia or as magnetically guidable drug transporters (Tietze et al., 2013; Unterweger et al., 2023). If SPIONs of a suitable size and surface coating are incubated with cells, they bind to their plasma membrane or are ingulfed by the cells, which can be used to label and track the cells in MRI. With SPIONs, lymphocytes and more recently, T cells carrying a chimeric antigen receptor (CAR-T cells) have been successfully tracked in MRI as they migrate through the body (Baeten et al., 2010; Liu and Li, 2014; Kiru et al., 2022). Besides tracking, the magnetic cargo also enables the steering of the cells when appropriate magnetic fields are applied. In magnetic cell culture, SPION-loaded cells have been magnetically arranged in patterns or in three-dimensional structures *in vitro* (Kappes et al., 2022). *In vivo*, SPION-loaded cells have already been magnetically accumulated in a target area. This has been exploited previously for several cell types such as dendritic cells, stem cells, and endothelial cells for tumor vaccination, to control tissue injury or application in regenerative medicine (Polyak et al., 2008; Tukmachev et al., 2015; Jin et al., 2016). In the fight against tumors, the magnetic functionalization and navigation of immune cells such as T cells or NK cells is under extensive research (Sanz-Ortega et al., 2019a; Sanz-Ortega et al., 2019b; Muhlberger et al., 2020; Boosz et al., 2021; Pfister et al., 2023). Previously, T cells have been loaded with SPIONs to enable their magnetic targeting for adoptive immune therapies such as CAR-T cell therapies.

SPIONs with citrate- or gold-coating were initially developed for cell labeling (Muhlberger et al., 2019; Stein et al., 2020). Citrate-coating rendered the particles biocompatible and despite their negative charge, particles were readily taken up by cells. The surface-binding property of gold, on the one hand, presents strong and simple binding motifs on gold nanoparticles (AU-NPs) e.g., for thiols or disulfide compounds. Amines or carboxylates on the other hand enable functionalization of the gold surface with antibodies or DNA aptamers (Ding and Liu, 2024). Caused by the coherent oscillation of free electrons, the so-called surface plasma resonance (SPR) effect, the optical property of AU-NPs expands their field of application for use as biosensors, imaging agents and photothermal agents for medical diagnosis, imaging and treatment (Yang et al., 2022). The combination of highly magnetic SPIONs and an easy-to-functionalize gold surface generates a hybrid carrier system (Au-SPIONs), to which medically useful substances can be attached (Stein et al., 2020).

One main hurdle in immune cell therapy is the immunosuppressive tumor microenvironment, which impairs recruitment of effector cells and mediates their inactivation (D'Aloia et al., 2018). Additional hurdles limiting the therapeutic success are various difficulties such as extravasation or ineffective trafficking of immune cells such as T cells to tumor sites (Hou et al., 2021). Besides T cells, engaging monocytes and macrophages as anti-tumoral effector cells appears very attractive, due to their capacity to overcome challenges associated with solid tumors such as infiltration, immunosuppression within the tumor microenvironment, lymphocyte exclusion, and target antigen heterogeneity (Ritchie et al., 2007; Gabrusiewicz et al., 2020; Klichinsky et al., 2020; Blumenthal et al., 2022; Goswami et al., 2023).

So far, CAR macrophages have shown potent anti-tumor activity in pre-clinical solid tumor models, and the anti-HER2-specific CAR macrophage CT-0508 has been evaluated in a Phase I trial (Reiss et al., 2022). To reduce time and costs of the therapy, CAR monocytes are under development, which need no time and money consuming differentiation phase (Gabitova et al., 2021). Also, the adoptive transfer of IFN-α-producing monocytes is under investigation, which previously inhibited metastasis in breast cancer mice by reversing the immune suppressive tumor microenvironment (Moi et al., 2018). Since CAR monocytes are potent effector cells, the risks associated with CAR-T cell therapy can also occur here, such as cytokine release syndrome (CRS), on-target/off-tumor toxicity or neurotoxicity. This demands for a balance to be found between efficacy and toxicity (Brudno and Kochenderfer, 2016).

Thus, the targeted enrichment of cells in the tumor area is desirable to increase efficacy and reduce systemic side effects in the periphery. Analogously to the previously published loading of T cells with SPIONs, we established the loading of monocytes with SPIONs to gain their magnetic steerability for adoptive therapy. In this study, we examined the effect of citrate-stabilized gold-coated or citrate-coated SPIONs on THP-1 cells concerning uptake, development of reactive oxygen species, biocompatibility, and magnetic accumulation.

## 2 Materials and methods

### 2.1 Particle synthesis and physicochemical characterization

SPION^Citrate^ were synthesized according to an adjusted protocol of Elbialy et al. (2015), Muhlberger et al. (2019). In summary, iron (III) chloride and iron (II) chloride in a molar ratio of 2:1 (Fe^3+^: Fe^2+^) were precipitated to iron oxide nanoparticles using a 25% ammonia solution under rapid stirring. After 10 min of reaction, a sodium citrate solution was added, the temperature of the liquid was raised to 90°C within 15 min and was held at 90°C for another 15 min to stabilize the resulting SPION^Citrate^. After cooling the particles to room temperature, they were magnetically washed from excess citrate five times using acetone. SPION^Citrate^ were dried from acetone and redispersed in deionized water.

SPION^Gold^ for subsequent gold-coating were synthesized accordingly with a difference in the washing procedure. The acetone washing steps were replaced with a dialysis treatment. The particles were given into 10 kDa dialysis tubes and dialyzed five times against 4.5 L of deionized water. Finally, the particles were ultrasonicated to fine-tune the hydrodynamic size to ∼80 nm for the following gold coating.

The SPION^Gold^ synthesis was performed following the route of Stein et al. (2020) with slight adaptations. In short, SPION^Citrate^ with a hydrodynamic size of 80 nm, instead of the previously reported 110 nm SPION^Citrate^ from Stein et al., were brought to a boil. Then, a HAuCl_4_ solution was quickly added to the SPION dispersion to precipitate gold onto the particles. After 15 min of reaction, a citrate solution was injected to stabilize the generated SPION^Gold^ and the particles were cooled to room temperature.

SPION^Citrate^ were sterilized by filtration through syringe filters with 0.2 µm pore size (Sartorius, Goettingen, Germany), while SPION^Gold^ were autoclaved for 30 min at 121°C and 2 bar of pressure. Both particle systems were stored under sterile conditions at 8°C until characterization and further use.

Subsequently, the SPIONs were analyzed for their size, magnetic susceptibility, and zeta potential as described previously (15). For the determination of the iron content, the suspension was diluted 1:25 in deionized H_2_O (produced in-house via the Merck Milli-Q^®^ Direct water purification system, Darmstadt, Germany) and dissolved in 65% nitric acid (Carl Roth, Karlsruhe, Germany), for analysis via atomic emission spectroscopy (AES), using the Agilent 4200 MP-AES (Agilent Technologies, Santa Clara, CA, United States) with an external standard iron solution of 1,000 mg/L (Bernd Kraft, Duisburg, Germany) as a basis for a calibration curve. Measurements were performed in triplicates at a wavelength of 371.993 nm and averaged.

The Z-Average, further called hydrodynamic size, of the particle systems was measured via dynamic light scattering (DLS) using a Zetasizer Nano (Malvern instruments, Worcestershire, United Kingdom). SPION^Citrate^ were diluted to an iron concentration of 50 μg/mL with deionized water, while SPION^Gold^ were diluted to 25 μg/mL for the analysis at 25°C.

The investigation of the zeta potential of SPION^Citrate^ and SPION^Gold^ was conducted using the Zetasizer Nano (Malvern instruments, Worcestershire, United Kingdom). For the measurement, SPION^Citrate^ and SPION^Gold^ dispersions with iron concentrations of 50 μg/mL and 25 μg/mL, respectively, were adjusted to a pH of 7.3 using minimal amounts of 10 mM HCl or 10 mM NaOH solutions.

The magnetic volume susceptibility of both SPION systems was measured using a magnetic susceptibility meter (MS2G, Bartington Instruments, Witney, UK). The iron concentrations of all samples were diluted to 1 mg/mL using deionized water in order to gain comparable values.

All characterizations were performed in triplicates and results are presented as the calculated mean value.

### 2.2 Cells and culture conditions

THP-1 monocytic cells were purchased from DSMZ (Braunschweig, Germany) and cultured in Gibco™ RPMI medium 1640 supplemented with 2 mmol/L glutamine, 100 U/mL penicillin-streptomycin, both from Thermo Fisher Scientific (Waltham, MA, United States), and 10% fetal calf serum (FCS, Biochrom, Berlin, Germany) under cell culture conditions.

### 2.3 Colloidal stability of SPIONs in THP-1 medium

To investigate their colloidal stability, SPION^Citrate^ and SPION^Gold^ (0, 20, 40 and 80 μg/mL) were incubated in 200 µL THP-1 medium overnight under cell culture conditions. The next day, the nanoparticles were either resuspended or not. Then, 100 µL supernatant of the not-resuspended SPIONs or the resuspended SPIONs were transferred to a separate 96-well-plate. Subsequently, the absorbance was measured in the SpectraMax iD3 (Molecular Devices, San José, CA, United States) from 230 nm to 750 nm in 10 nm steps. Additionally, Eppendorf tubes with SPION^Citrate^ or SPION^Gold^ (0, 20, 40 and 80 μg/mL) in THP-1 medium were incubated overnight and the nanoparticle sedimentation was photographed on the next day.

### 2.4 Cellular uptake

To evaluate the particle uptake, 2 × 10^5^ THP-1 cells per mL medium were incubated in a 24-well plate with SPION^Citrate^ or SPION^Gold^ at an iron concentration of 20, 40 or 80 μg/mL, using the same volume of H_2_O or 2% DMSO as controls. After 24 h, cells were stained with 1 μL/mL Annexin V-FITC (AxV-FITC, ImmunoTools, Friesoythe, Germany) and 66.6 ng/mL propidium iodide (PI, Sigma-Aldrich, Taufkirchen, Germany) in Ringer’s solution (Fresenius Kabi, Bad Homburg, Germany). The side scatter, which describes the granularity of the cell and correlates with particle uptake, was measured with a Gallios flow cytometer and analyzed with Kaluza (1.3., 2.1) Analysis Software (both from Beckman Coulter, Brea, CA, United States).

Furthermore, the cellular iron content was quantified by AES. Therefore, 2 × 10^5^ THP-1 cells per mL medium were seeded in triplicates in a 24-well plate. The cells were either incubated with SPION^Citrate^ or SPION^Gold^ at an iron concentration of 20, 40 or 80 μg/mL, using the same volume of H_2_O or 2% DMSO as controls. After 24 h incubation, cells were washed with phosphate-buffered saline (PBS) and centrifuged at 300 g for 5 min to remove residual particles. Then, the cells were resuspended in PBS and counted by a MUSE Cell Analyzer. Cell pellets were formed by centrifugation at 10,000 g for 5 min. The pellets were dried at 300 rpm at 95°C for 30 min and lysed with 65%-nitric acid at 300 rpm at 95°C for 15 min. The samples were diluted with water and the iron content was measured with Agilent 4200 MP-AES.

### 2.5 Transmission electron microscopy

To visualize particle uptake and adherence on the cell surface transmission electron microscopy (TEM) was used. Therefore, 7 × 10^6^ THP-1 cells in 10 mL medium were incubated with 20 or 40 μg/mL of SPION^Citrate^ or SPION^Gold^ using H_2_O as control. After 24 h cells were washed and then fixed in 2.5% glutaraldehyde (Carl Roth, Karlsruhe, Germany) in 0.1 M PO_4_ buffer (Carl Roth) for 4 h. Afterwards cells were washed 3 times with 0.1 M PO_4_ buffer and kept at 4°C overnight. The next day, cells were stained with 1% osmium tetra oxide (OsO_4_) in 3% potassium ferricyanide (K3(Fe(Cn)6), (Science Service, Munich, Germany) for 2 h at room temperature and then washed with 0.1 M PO_4_ buffer overnight at 4°C. Subsequently, cells were transferred into a matrix of 2% agarose (low melting point, Science Service) in 0.1 M PO_4_ buffer overnight using an Eppendorf reaction tube. After being dehydrated in agarose through an ethanol step, the cells were finally transferred to Epon using an acetone-epon mixture and left to polymerize for 48 h at 60°C. With the Leica Ultracut UCT (Wetzlar, Germany) ultra-thin sections with a thickness of around 50 nm of the samples were prepared and put on copper grids (Science Service). The received sections were contrasted with lead citrate for 10 min followed by uranyl acetate (Serva, Heidelberg, Germany) for additional 10 min. For the images the TEM 906 LEO (Zeiss, Oberkochen, Germany) with a CCD-camera residual light amplifier from TRS Tröndle (Moorenweis, Germany) was used and further the ImageSP SysPROG software. An acceleration voltage of 80 kV and a magnification of 12,930-fold were used for image taking.

### 2.6 Reactive oxygen species assay

To determine the generation of oxidative stress in THP-1 cells by nanoparticles, THP-1 cells were stained with 20 µM 2′,7′-Dichlorodihydrofluoresceindiacetat (DCFH, Sigma Aldrich) and incubated under cell culture conditions for 30 min. Cells were washed, seeded in a concentration of 1.8 × 10^5^ THP-1 cells per mL medium in a 96-well plate, and incubated with either SPION^Citrate^ or SPION^Gold^ at a concentration of 20, 40 or 80 μg/mL, with H_2_O as negative control and 2 mM H_2_O_2_ or 2% DMSO as positive controls. After 3 h, 6 h and 24 h, 50 µL of cells were stained with 66.6 ng/mL PI, 1 µl/ml Annexin V-APC (AxV, ImmunoTools, Friesoythe, Germany) and 30 µM monobromobimane (MBB, Sigma Aldrich) in Ringer’s solution at 4°C under light protection and analyzed in flow cytometry.

### 2.7 Magnetic accumulation of SPION-loaded THP-1 cells

THP-1 cells were incubated with 0, 20, 40 or 80 μg/mL SPION^Citrate^ or SPION^Gold^ for 24 h and subsequently stained with 20 nM Hoechst 33342 (Hoe, Thermo Fisher Scientific, Waltham, MA, United States). µ-Slides I Luer with a channel height of 0.4 mm (Ibidi, Gräflingen, Germany) were used for microscopy. The slides were coated with FCS to prevent THP-1 cells from adhering to the channel surface. A NdFeB disc-shaped magnet (S-15-08-N, Webcraft GmbH, Gottmadingen, Germany) was used for magnetic attraction with 15 mm diameter, 8 mm height, N42 magnetization, Ni-Cu-Ni coating, and axial direction of magnetization.

Slides without magnet were used as controls for each SPION-concentration. Immediately after the magnet was placed onto the slide, cells were pipetted in the slide channel at the opposite side. After 10 min, the slide was analyzed with Zeiss Axio Observer Z1 fluorescence microscope (Carl Zeiss AG, Oberkochen, Germany). Pictures were taken below the center of the magnet, on the edge of the magnet and then in 5 mm increments up to 3 cm away from the magnet and analyzed with ImageJ software (version 1.52a).

Further, we confirm cellular viability in the presence of an external magnetic field (EMF). To do so, THP-1 cells were seeded in a concentration of 2 × 10^4^ cells per well in 96 well plates. The cells were incubated with 0, 20, 40 or 80 μg/mL SPION^Citrate^ or SPION^Gold^ for 24 h, washed and subsequently enriched by placing a NdFeB disc-shaped magnet underneath each well (S-15-08-N, Webcraft GmbH, Gottmadingen, Germany; 15 mm diameter, 8 mm height, N42 magnetization, Ni-Cu-Ni coating, and axial direction of magnetization). Cells were exposed to the EMF for 24 h and stained with 1 μL/mL Annexin V-APC (AxV-APC, ImmunoTools, Friesoythe, Germany), 66.6 ng/mL PI in Ringer’s solution at 4°C under light protection and analyzed in flow cytometry. Viability was measured with a Gallios flow cytometer and analyzed with Kaluza (1.3., 2.1) Analysis Software (both from Beckman Coulter, Brea, CA, United States).

### 2.8 Computational model of the magnetic field, force and accumulation of SPION-loaded cells

We validated our experiments with a computational model. For the disc-shaped magnet used in the experiments, analytical expressions allow efficient computation of the magnetic field and the magnetic force on the SPION-loaded cells. We computed the magnetic field using the analytical expression presented by Derby and Olbert (2010). For the magnetic force on the SPION-loaded cells, we used the analytical expression derived by Wirthl et al. (2024), including a linear magnetization model with saturation magnetizations as measured by Stein et al. (2020).

Additionally, we computed the magnetic accumulation of the SPION-loaded cells. The force acting on a cell is the sum of the magnetic force $F_{mag}$ and the drag force $F_{drag}$ . Based on Stokes’ law, the drag force is the resistive force exerted by the fluid on the cell, opposing its motion. Hence, the movement of the cell based on Newton’s second law of motion is given by

$$F_{mag}+F_{drag}=ma=m\frac{dv}{dt}$$

with m being the mass of the cell, v its velocity and a its acceleration. We used the backward Euler method to solve this ordinary differential equation for the cell velocity (Burden and Faires, 2015), which subsequently allowed us to compute the trajectory of the SPION-loaded cells over time.

### 2.9 Data analysis and statistics

Data were processed in Microsoft Excel (Microsoft, Redmond, WA, United States). Statistical analysis and graph creation were performed with GraphPad PRISM 9.0.2 from Graph-Pad Software, Inc. (San Diego, CA, United States). Data are shown as the mean ± standard deviation (SD) with at least three replicates, unless otherwise specified. Statistical p-values ≤ 0.05 were considered as statistically significant.

## 3 Results

### 3.1 Physicochemical nanoparticle characterization

Hydrodynamic size, zeta potential at pH 7.3 and magnetic volume susceptibility of three (SPION^Citrate^) or two (SPION^Gold^) independently synthesized batches of SPIONs were investigated (Table 1). SPION^Citrate^ showed a hydrodynamic diameter of 52 nm ± 2 nm with a polydispersity index (PDI) of 0.15 ± 0.01 while SPION^Gold^ were 120 nm ± 0 nm with a PDI of 0.17 ± 0.01, respectively. For both SPION types, the size distribution was quite narrow. With −48.8 mV ± 6.2 mV and −41.6 mV ± 8.9 mV, SPION^Citrate^ and SPION^Gold^ showed comparable zeta potentials and a strong electrostatic stability. The magnetic susceptibility of SPION^Citrate^ ((4.18 ± 0.04) ⋅ 10^−3^) was slightly lower than that of SPION^Gold^ ((4.95 ± 1.59) ⋅ 10^−3^). The increased susceptibility of SPION^Gold^ is caused by the higher susceptibility of SPION^Citrate^ batches used as a basis for the gold-coating ((5.34 ± 1.60) ⋅ 10^−3^). The gold coating therefore reduced the susceptibility of SPION^Gold^ to 92.7% ± 2.1% of the initial value of primary SPIONs.

**TABLE 1:** Physicochemical characterization of SPION^Citrate^ and SPION^Gold^.

Physicochemical features	SPION^Citrate^	SPION^Gold^
Hydrodynamic diameter in H_2_O (nm)	52 ± 2	120 ± 0
PDI (a.u.)	0.15 ± 0.01	0.17 ± 0.01
Zeta potential at pH 7.3 (−)	−48.8 ± 6.2	−41.6 ± 8.9
Magnetic susceptibility •10^−3^ for 1 mg Fe/mL (a.u.)	4.18 ± 0.04	4.95 ± 1.59

**FIGURE 1:**
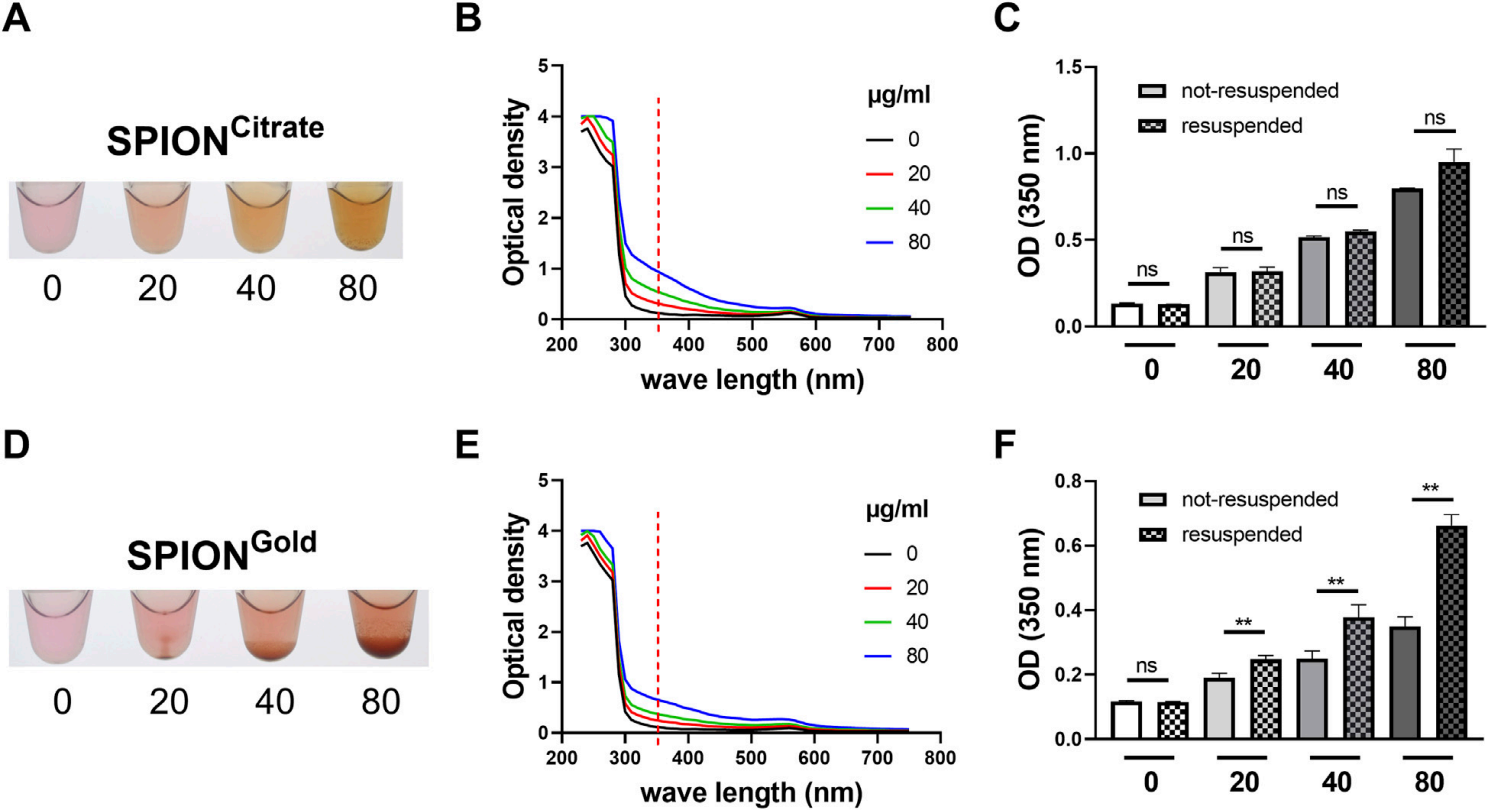
Colloidal stability of SPIONs in cell culture medium. SPION^Citrate^ **(A–C)** and SPION^Gold^ **(D–F)** were incubated in concentrations of 20, 40, 80 μg/mL overnight in THP-1 cell culture medium. Medium without nanoparticles served as controls. **(A, D)** Macroscopic pictures of sediments after overnight incubation. **(B, E)** Spectrum (250 nm–750 nm) of SPIONs diluted in cell culture medium. **(C, F)** Optical density at 350 nm after overnight incubation of the samples. Comparison of not-resuspended and resuspended SPIONs. Shown are the mean values with standard deviation. Significances were calculated using unpaired t-test with Welch’s correction; *p ≤ 0.05; **p ≤ 0.01. ns, not significant.

The colloidal stability of nanoparticles influences their cellular uptake and toxicity. To determine the colloidal stability of SPION^Citrate^ and SPION^Gold^ in THP-1 cell culture medium, the nanoparticles were incubated overnight to eventually let them agglomerate and sediment. Especially the SPION^Gold^ particles showed a macroscopically dose-dependent tendency for sedimentation. In contrast, sedimentation of SPION^Citrate^ particles was less pronounced (Figures 1A, D).

The measured spectrum (230–750 nm) of the nanoparticle dilutions showed an optimal wavelength to detect the SPIONs at 350 nm (Figures 1B, E). To analyze the colloidal stability of SPIONs, the samples were either resuspended or left not-resuspended, supernatants were taken and the optical densities (ODs) were compared. When nanoparticles are colloidally stable, the absorption of supernatants from not-resuspended and resuspended samples should be the same. As expected, SPION^Citrate^ and SPION^Gold^ displayed a dose-dependent increase in the absorption. SPION^Citrate^ showed only a slight difference in the OD values of the supernatants of not-resuspended and resuspended samples. SPION^Gold^, on the other hand, showed a significant difference between the absorption values already at 20 μg/mL SPIONs (Figures 1C, F), indicating nanoparticle sedimentation. Thus, in direct comparison, SPION^Citrate^ showed a better colloidal stability than SPION^Gold^ under the tested conditions.

### 3.2 Cellular uptake of SPION^Citrate^ and SPION^Gold^

In order to control SPION-loaded THP-1 cells within a magnetic field, the cells require a sufficiently high loading with nanoparticles. Thereby their magnetic controllability is independent of whether the cells take up the particles during loading or whether the particles attach to the cell surface. Three different methods were used to assess the accumulation of SPION^Citrate^ and SPION^Gold^ in and/or on the THP-1 cells.

**FIGURE 2:**
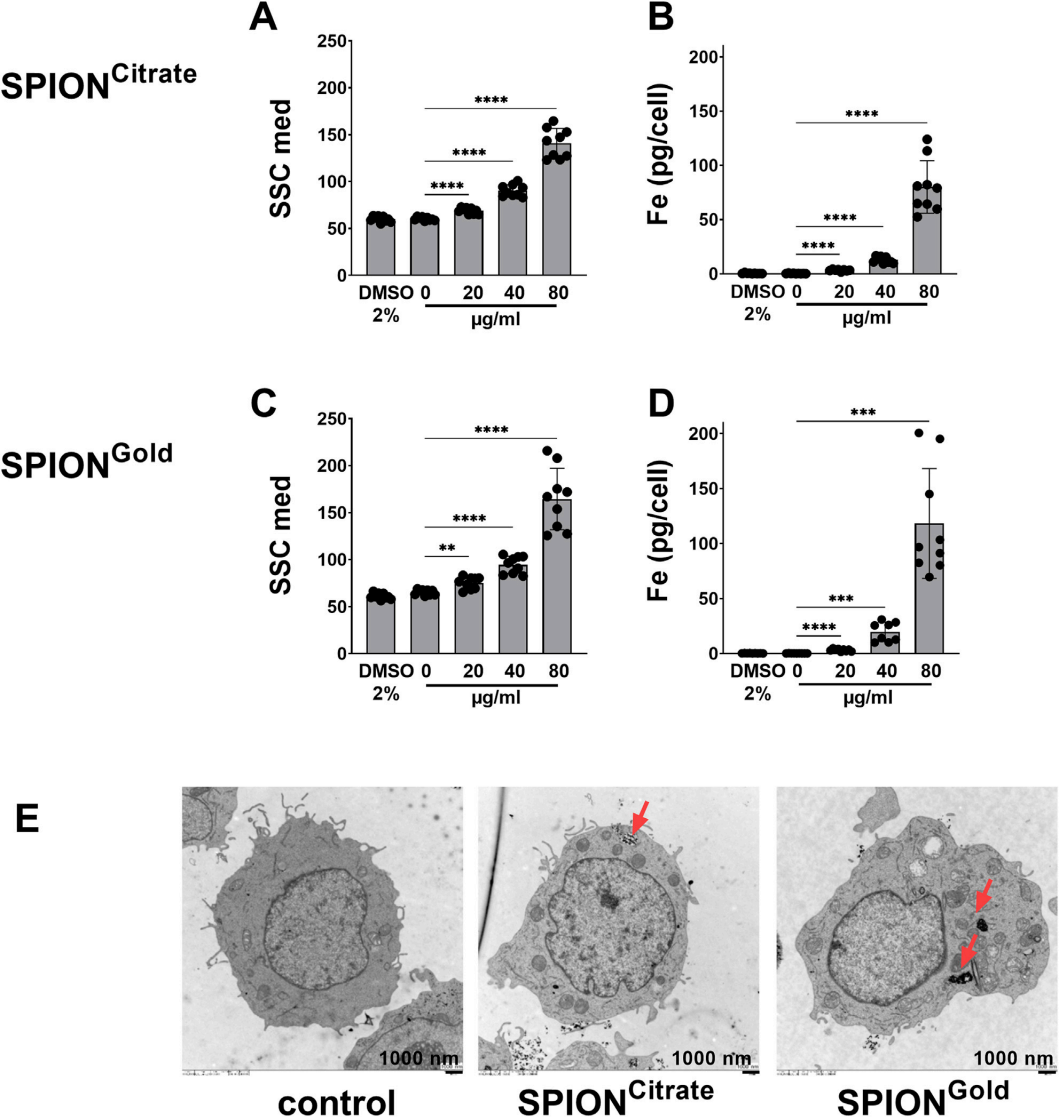
Cellular uptake of SPION^Citrate^ and SPION^Gold^. THP-1 were incubated with 20, 40 or 80 μg/mL SPION^Citrate^ **(A, B)** or SPION^Gold^ **(C, D)** for 24 h. DMSO served as toxicity control while H_2_O (0 μg/mL) served as a negative control. **(A, C)** Side Scatter (SSC) increase of AxV-FITC-/PI-cells in flow cytometry shows nanoparticle uptake. **(B, D)** Iron amount per cell was determined by atomic emission spectroscopy. **(A–D)** Shown are the mean values with standard deviation. Significances were calculated using unpaired t-test with Welch’s correction; **(E)** Transmission electron microscopy of THP-1 cells incubated with 40 μg/mL SPION^Citrate^ or 20 μg/mL SPION^Gold^. H_2_O served as a control. Arrows indicate SPIONs in intracellular vesicles. *p ≤ 0.05; **p ≤ 0.01, ***≤0.001; ****≤0.0001; ns, not significant.

First, the side scatter properties of the cells were assessed by flow cytometry. Cells interacting with nanoparticles show an increase in granularity. It should be noted that cell morphology is also influenced by cell death processes, thus gating on viable cells is necessary to exclude cells with morphological alterations caused by dying processes such as blebbing or membrane rupture. Therefore, we analyzed only cells with intact plasma membrane (AxV-FITC-/PI-) to analyze cellular side scatter alterations caused by the SPIONs. When cells were incubated with SPIONs for 24 h, THP-1 cells showed a significant increase in cell granularity already at a concentration of 20 μg/mL, which increased dose-dependently (Figures 2A, C).

To quantify the iron content in the cells, THP-1 cells incubated with SPIONs for 24 h were washed to remove free nanoparticles. Subsequently, the cell number was determined and the iron content of the cell pellets was measured in AES. Again, significant amounts of cellular iron were already detected when cells were incubated with 20 μg/mL SPIONs. When incubated with increasing amounts of nanoparticles, the cellular iron content increased in a dose dependent manner, which was in line with the SSC measurements. When comparing the iron amounts per cell, a large increase was detected from 40 μg/mL to 80 μg/mL for both particle types, which is due to the fact, that the particles agglomerated and strongly sedimented in concentrations of 80 μg/mL, so that at the bottom of the wells, where the cells are growing, more particles arrived than nominally administered (Figures 2B, D).

For visualization of the nanoparticle localization TEM was performed. Internalization into intracellular vesicles was visible for both SPION types. When cells were incubated with 40 μg/mL SPION^Citrate^, single particles were detected in intracellular vesicles, while with 20 μg/mL SPION^Gold^ no individual particles could be identified which was probably due to increased agglomeration of this type of nanoparticle (Figure 2E, see red arrows). Attachment of particles to the plasma membranes was detected as well.

### 3.3 Reactive oxygen species assay

To assess the generation of reactive oxygen species (ROS) by Fenton reactions leading eventually to cell death, THP-1 cells were incubated with 20, 40 or 80 μg/mL SPIONs and analyzed after 3, 6 and 24 h after particle incubation. H_2_O served as negative control, H_2_O_2_ and DMSO served as positive controls for ROS or general toxicity, respectively. All values were standardized to the H_2_O incubated cells.

DCFH was used as an indicator for general ROS. Non-polar DCFH-DA is taken up by the cells and converted by esterases into polar DCFH. Subsequently, DCFH is oxidized by ROS to fluorescent DCF (Rastogi et al., 2010). MBB served as indicator of the redox status of glutathione (GSH). As an antioxidant, GSH serves as an electron donor and is oxidized to glutathione disulfide (GSSG) in the presence of ROS. MBB reacts with thiols in non-oxidized GSH and shows strong fluorescence. As soon as GSSG occurs, the fluorescence is decreased (Cossarizza et al., 2009). PI discriminates between cells with intact (PI-) and disturbed plasma membranes (PI+).

**FIGURE 3:**
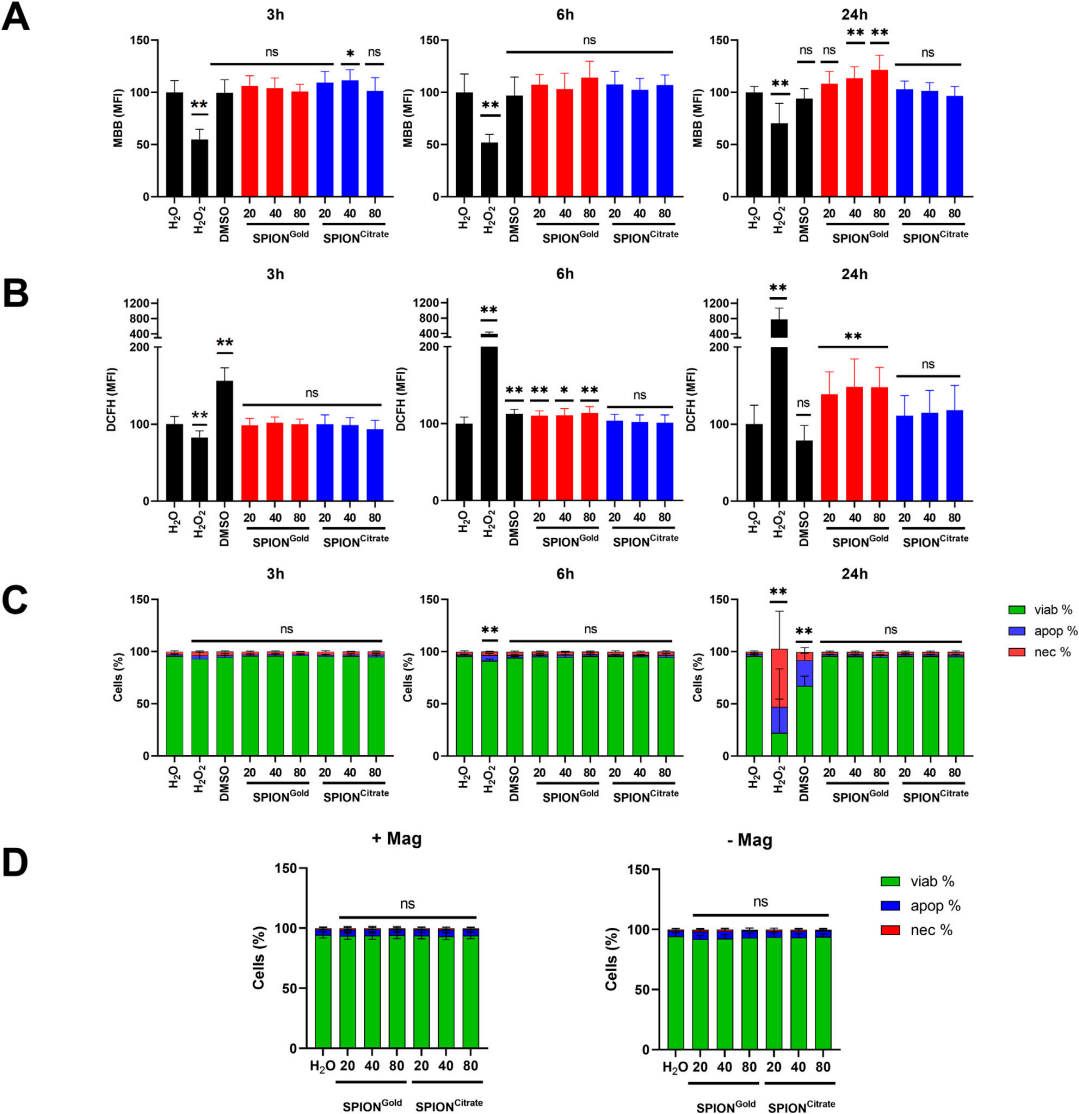
Influence of SPIONs on cellular ROS generation and viability. THP-1 cells were incubated with 20, 40, or 80 μg/mL SPION^Citrate^ or SPION^Gold^ and analyzed after 3, 6 and 24 h. H_2_O served as negative control, H_2_O_2_ and DMSO as positive controls for ROS or toxicity. The negative control was set to 100% and the treated samples were calculated accordingly. **(A)** Amount of reduced glutathione was determined by staining with MBB. **(B)** Intracellular general ROS was analyzed by staining with DCFH. **(C, D)** Cell viability of SPION^Gold^- and SPION^Citrate^-loaded cells was determined in the absence **(C)** and in the presence **(D)** of an EMF by staining with AxV and PI. AxV-/PI- cells are considered viable, AxV+/PI- apoptotic, and PI+ necrotic. Shown are the mean values of three independent experiments performed in triplicates with standard deviations. Significances were calculated using unpaired t-test with Welch’s correction; *p ≤ 0.05; **p ≤ 0.01. ns, not significant.

When incubating the cells with H_2_O_2_ as positive control, ROS is generated which is indicated by loss of MBB fluorescence and increase in DCF fluorescence. After 3 h, there was hardly oxidative stress detectable in the SPION treated samples, no matter if cells were stained with MBB or DCFH. After 6 h, in the SPION^Gold^ treated cells general ROS was increased as reflected by DCF fluorescence. After 24 h, this effect has been further increased in SPION^Gold^ treated cells, while no DCF fluorescence was measurable in SPION^Citrate^ treated ones. Interestingly, MBB fluorescence was dose-dependently elevated in SPION^Gold^ treated cells, which indicates an attempt by the cells to upregulate the antioxidant systems in order to cope with the ROS (Figures 3A, B). When analyzing the toxicity of SPION^Gold^ and SPION^Citrate^, we did not find a decrease in viability at any of the three measurement time points, while H_2_O_2_ and DMSO induced cell death in a time dependent manner (Figure 3C). Additionally, we investigated the effect of an EMF on cell viability alone and in combination with SPION^Citrate^ and SPION^Gold^. After exposing non-loaded and loaded THP-1 cells to an EMF for 24 h we did not observe any significant alteration in cell viability (Figure 3D). We conclude that the ROS induced by SPION^Gold^ was neutralized by the cells by upregulation of the antioxidant systems so that both types of nanoparticles did not decrease cell viability.

### 3.4 Magnetic accumulation

**FIGURE 4:**
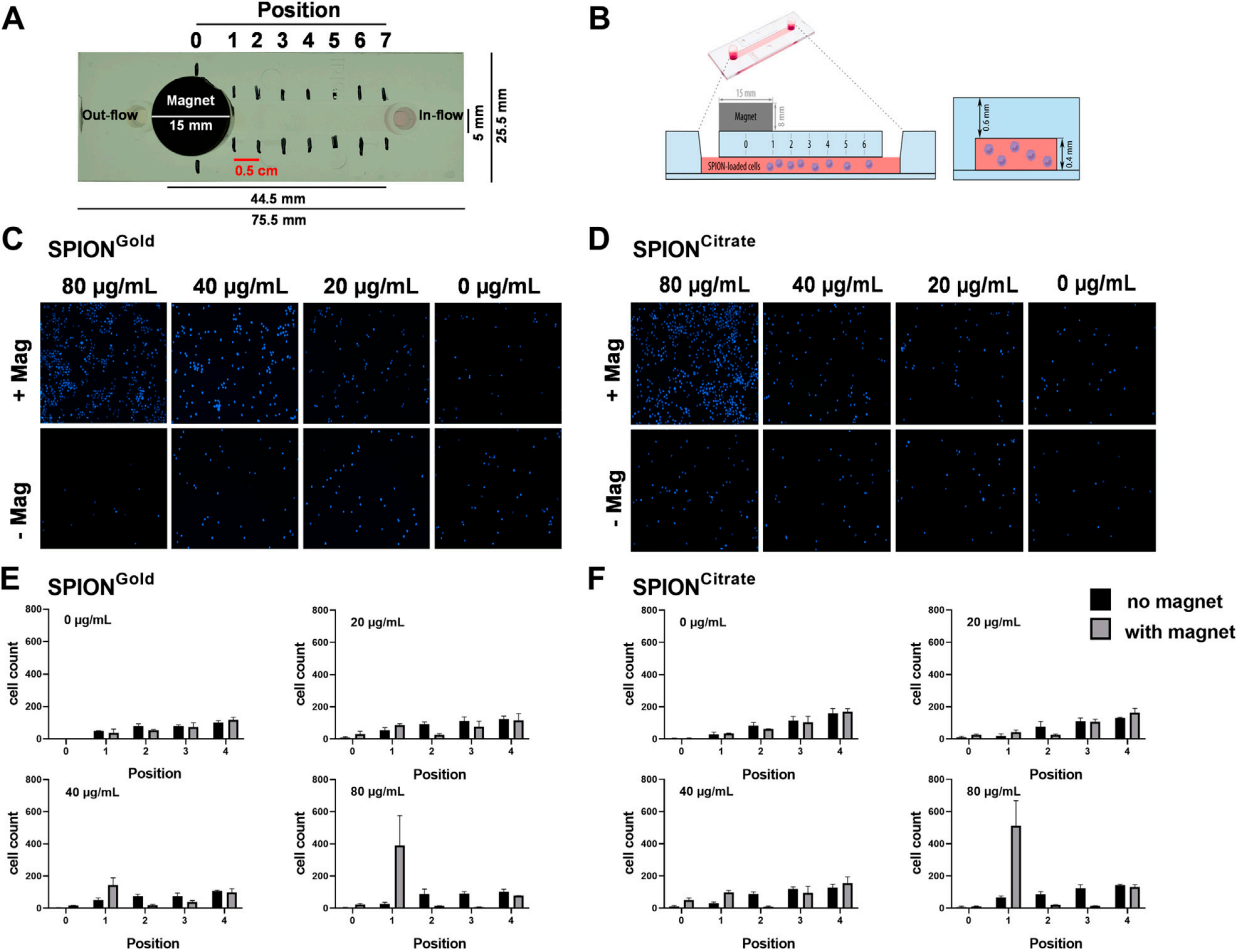
Experimental determination of magnetic enrichment of SPION-loaded THP-1 cells. THP-1 cells were incubated for 24 h with SPION^Citrate^ or SPION^Gold^. After washing, cells were transferred into Ibidi µ-Slides with a magnet placed on the channel. Cells were allowed to accumulate for 10 min. Thereafter, respective fluorescence images were captured which allowed to quantify cell numbers. **(A)** Photo of the Ibidi flow slide with magnet from above. **(B)** Schematic sketch of the experimental setup (proportions not to scale). **(C, D)** Fluorescent microscopic pictures of the Hoe-stained cells. **(E, F)** Cell count determined from the fluorescent microscopic pictures. Experiment was performed in triplicates. Shown are the mean values and standard deviations.

The magnetic controllability of THP-1 cells after SPION-loading was evaluated under the influence of a magnetic field in Ibidi µ-Slides. A magnet was placed onto the channel on one side, while SPION-loaded Hoe-stained cells were added on the opposite side of the slide. Then, the slides were kept at room temperature for 10 min and subsequently the localization of the cells analyzed via fluorescence microscopy. Unloaded cells and slides without magnet served as controls. The experimental setup is shown as photography from above and as model from the side in Figures 4A, B.

When cells without particle loading were used, they were not magnetically attracted. In contrast, cells loaded with 80 μg/mL SPION^Citrate^ or SPION^Gold^ showed a clear accumulation at the edge of the magnet (position 1). For conditions with less particles, only 40 μg/mL SPION^Gold^ enabled a weak accumulation towards the edge of the magnet. Within half a centimeter distance from the edge of the magnet, for both SPION types the cell number was visibly reduced in the immediate vicinity (position 2) because the cells centered at the point of strongest attraction (position 1). If the distance of the cells exceeded 1 cm from the magnet, the cell counts become increasingly similar to the measurements of the controls (Figures 4C–F).

**FIGURE 5:**
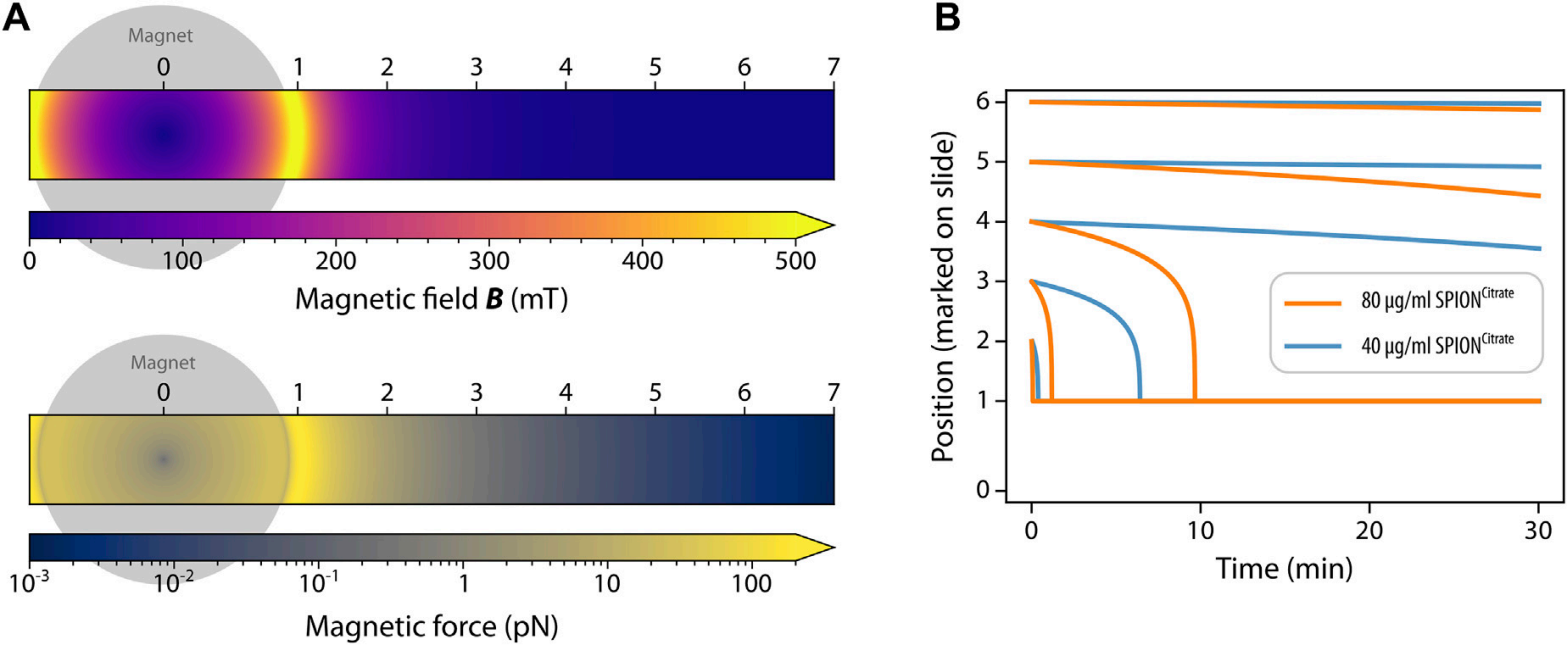
Simulation of the magnetic field, force and movement of cells. **(A)** Magnetic field (top) and force (bottom). **(B)** Movement of SPION^Citrate^ -loaded cells over time. For all simulations, we assumed a vertical distance of 0.6 mm between the magnet and the cells.

Based on the magnetic properties of the disc magnet and the SPION-loaded cells, we simulated the magnetic field and force with the result depicted in Figure 5. The strongest magnetic field occurs directly below the edge of the magnet (Figure 5A top). Similarly, the strongest magnetic force occurs below the edge of the magnet, while the magnetic force rapidly decreases with distance, so that the force at the inflow is five orders of magnitude smaller (Figure 5A bottom). Figure 5B shows the simulated movement of SPION^Citrate^ -loaded cells over time. We compared the movement of cells loaded with 40 μg/mL and with 80 μg/mL SPION^Citrate^, which were initially located in different positions as marked on the slide (see Figure 4A). For both concentrations, cells initially located within 1 cm of the magnet (Positions 2 and 3) accumulated around the magnet within minutes. At greater distances, the magnetic force diminishes, causing minimal cell movement. Overall, the computer simulation results agree with the experimental findings presented in Figure 4. Both the experiment and simulation consistently demonstrated that effective magnetic control of SPION-loaded cells is feasible only within a short range from the magnet.

The agreement between experimental results and simulations validates the computational model for future use in estimating the potential for magnetic enrichment of cells under specific conditions. More broadly, a comprehensive computational model will facilitate a more systematic and faster design space exploration than relying solely on experimental methods.

## 4 Discussion

The loading of cells with SPIONs enables their control by an external magnetic field. We previously established the loading of T cells with citrate-coated SPIONs. Here we have shown that THP-1 cells can also be loaded with SPIONs, allowing their magnetic accumulation under experimental conditions. For magnetic control, the cells must be sufficiently loaded with SPIONs, without compromising the cells viability.

With increasing concentration, nanoparticles might agglomerate and sediment, altering their biological effects. When we investigated the colloidal stability of SPION^Citrate^ and SPION^Gold^, we found that SPION^Citrate^ were colloidally stable under the used conditions, while SPION^Gold^ started to agglomerate and therefore sediment at a concentration as little as 20 μg/mL (Figure 1). This can be due to the difference in their size since gravitational sedimentation of nanoparticles is greatly impacted by their size (SPION^Citrate^: 52 ± 2 nm; SPION^Gold^: 120 ± 0 nm). Ultrasmall particles (<10 nm) distribute by diffusion, while larger particles (>100 nm) tend to sediment (Hinderliter et al., 2010). This was previously also shown for gold nanoparticles (Alexander et al., 2013). For our experimental set up we used cell culture medium supplemented with 10% FCS, which stabilizes nanoparticles, due to the formation of a protein corona (Allouni et al., 2009; Wiogo et al., 2011). The positive effect of the formation of a protein corona on the colloidal stability was previously investigated by Boosz et al. (2021). Here, they showed that SPION^Citrate^, which are also used in this current study, were colloidally stable in cell culture medium containing 10% FCS, but a reduction of the FCS concentration to 2% led to agglomeration and sedimentation (Boosz et al., 2021). Although the iron uptake was higher when using 2% FCS, the cell number was strongly reduced. Similar results were obtained by Mahmoud et al. (2020) in their study of gold nanorods, where the addition of fetal bovine serum improved colloidal stability, while nanoparticles aggregated in serum-free medium.

Nanoparticle sedimentation can have an impact on the uptake and viability of cells since the sedimenting particles cause a higher concentration on the cells than the specified nominal dose (Wittmaack, 2011).

Stein et al. (2020) also investigated the cellular uptake of Cit-Au-SPIONs. Here, Jurkat cells showed a statistically significant particle uptake only with a high iron concentration of 100 μg/mL. Since T cells are non-phagocytosing cells, they take up only low amounts of iron. In this study however, we used monocytes as model cells. As professional phagocytic cells, monocytes are responsible for the detection, phagocytosis and clearance of potentially hazardous organisms, apoptotic cells, insult-related debris and metabolic byproducts, thus efficient uptake of nanoparticles was expected as well (Christofides et al., 2022). Indeed, in our hands, THP-1 cells took up SPIONs significantly, already at an iron concentration of 20 μg/mL (Figure 2). This was in line with findings of Karimian-Jazi et al. (2020) who showed that nanoparticles are mainly taken up by circulating monocytes and tumor macrophages and less by tumor or T cells. Based on our sedimentation data, the increased uptake for SPION concentrations of 80 μg/mL can be ascribed to agglomeration and sedimentation. In the AES measurements, also artefacts caused by insufficient removal of particles during the washing steps are expected to be responsible for high SPION concentrations.

Higher nanoparticle uptake often leads to cytotoxicity as shown previously (Lesniak et al., 2012). Thus, we needed to find a balance between uptake and toxicity. In our case, iron released from the SPIONs can cause oxidative stress. After monocytes have taken up SPIONs, the particles enter the lysosomes and the iron oxide is degraded into iron ions. Via the Fenton and Haber-Weiss reaction, the free iron causes increasing reactive oxygen species, which can cause oxidative stress, mitochondrial dysfunction, DNA destruction and inflammation (Polasky et al., 2022). ROS generation and therefore oxidative stress were only measurable in SPION^Gold^ -loaded cells. A dose-dependent increase of reduced glutathione was shown after 24 h in SPION^Gold^ -loaded cells as a result of upregulation of the antioxidant system over time. In our experiments, THP-1 cells remained viable in all tested conditions and concentrations for 24 h for SPION^Gold^ as well as SPION^Citrate^. ROS induced by SPIONs were obviously efficiently detoxified (Figure 3). Previously, Stein et al. (2020) tested the biocompatibility of SPION^Gold^ used on Jurkat cells. No cell toxicity was observed after 48 h until 100 μg/mL. Also, SPION^Citrate^ showed a good biocompatibility in experiments before with Jurkat cells, murine, or human primary T cells (Muhlberger et al., 2019; Muhlberger et al., 2020; Boosz et al., 2021; Pfister et al., 2023).

The aim of our experiments was to find out if SPION^Citrate^ or SPION^Gold^ can be used for the magnetization of monocytes in order to magnetically control them for later use in immune therapy. Previously, it had been investigated if cell loading with SPION^Citrate^ enabled their magnetic control under static or dynamic conditions. SPION^Citrate^-loaded T cells containing an iron amount of 1 pg/cell were attracted in various experimental settings with a peristaltic pump (Boosz et al., 2021; Behr et al., 2022). Kappes et al. (2022) used SPION^Citrate^ and alternatively coated SPIONs for the magnetization of primary fibroblasts for use in magnetic cell culture. Here, we investigated the magnetic enrichment of SPION-loaded THP-1 cells under an experimental artificial setting using Ibidi slides with a continuous channel. Under these conditions, we found that cells which had been loaded with 80 μg/mL SPIONs were sufficiently magnetically attractable (Figure 4). The data achieved experimentally fit the data obtained by simulations (Figure 5). Whether this effect is sufficient for magnetic enrichment in the body is unclear and has to be investigated in the future.

## Data Availability

The raw data supporting the conclusions of this article are available from the authors on request.
